# *Bifidobacterium longum* subsp. *infantis* regulates Th1/Th2 balance through the JAK-STAT pathway in growing mice

**DOI:** 10.20517/mrr.2023.64

**Published:** 2024-01-19

**Authors:** Mengfan Ding, Bowen Li, Haiqin Chen, R. Paul Ross, Catherine Stanton, Shilong Jiang, Jianxin Zhao, Wei Chen, Bo Yang

**Affiliations:** ^1^State Key Laboratory of Food Science and Resources, Jiangnan University, Wuxi 214122, Jiangsu, China.; ^2^School of Food Science and Technology, Jiangnan University, Wuxi 214122, Jiangsu, China.; ^3^International Joint Research Center for Probiotics & Gut Health, Jiangnan University, Wuxi 214122, Jiangsu, China.; ^4^APC Microbiome Ireland, University College Cork, Cork T12 R229, Ireland.; ^5^Teagasc Food Research Centre, Moorepark, Co. Cork P61 C996, Ireland.; ^6^Nutrition and Metabolism Research Division, Innovation Center, Heilongjiang Feihe Dairy Co., Ltd, Beijing 100015, China.; ^7^PKUHSC-China Feihe Joint Research Institute of Nutrition and Healthy Lifespan Development, Beijing 100083, China.; ^8^National Engineering Research Center for Functional Food, Jiangnan University, Wuxi 214122, Jiangsu, China.

**Keywords:** *Bifidobacterium longum* subsp.* infantis*, Th1/Th2 balance, JAK/STAT pathway, gut microbiota, bifidobacterial community

## Abstract

**Objectives:** *Bifidobacterium longum* subsp. *infantis* is a dominant bacterium in infant gut, which plays a critical role in maintaining the health and development of infants. This study investigated the abilities of eight different strains of *B. longum* subsp. *infantis* to regulate the T helper (Th)1/Th2 balance.

**Methods:** Eight *B*. *longum* subsp. *infantis* strains, including I2MI (FJSWXI2MIM1), I4MI [FJSWXI4MI (CCFM1270)], I4MNI (FJSWXI4MNIM1), I5TI (FJSWXI5TIM1), I6TI (FJSWXI6TIM1), I8TI [FJSWXI8TI (CCFM1271)], I10TI [FJSWXI10TI (CCFM1272)], and B6MNI [BJSWXB6MNIM1 (CCFM1269)], were gavaged to BALB/C pups in both female (*n* = 8) and male (*n* = 8) mice starting from 1 to 3 weeks old (1 × 10^9^ CFU/day/mice). Selected immune cells were assessed by immunofluorescence and flow cytometry. Cytokines and immunoglobulins were determined by ELISA. Bacterial and bifidobacterial communities were determined by *16S rRNA* gene sequencing and bifidobacterial *groEL* sequencing.

**Results:** *B*. *longum* subsp. *infantis* I4MI and I8TI were shown to increase the ration of colonic IgG2a/IgE in male mice (*P* < 0.05). B6MNI was demonstrated to significantly increase the levels of colonic IFN-γ and IgG2a, as well as the ratio of IgG2a/IgE in female mice (*P* < 0.05). It was also shown to significantly increase the ratio of colonic IgG2a/IgE (*P* < 0.05) and reduce the level of colonic IL-4 in male mice (*P* < 0.05). Furthermore, B6MNI was demonstrated to regulate colonic JAK/STAT pathway in both male and female mice. I4MI, I5TI, and B6MNI were shown to increase the relative abundance of *Bifidobacterium* and *B. longum* subsp. *infantis* in both male and female mice, whereas I8TI was only shown to increase the relative abundance of *Bifidobacterium* and *B. longum* subsp. *infantis* in male mice (*P* < 0.05).

**Conclusion:** These results indicated supplementation with *B. longum* subsp. *infantis* in early infancy may regulate the Th1/Th2 immune balance, which may prevent the development of related diseases.

## INTRODUCTION

With the rising incidence of various non-infectious diseases, there has been intense interest in discovering potential causes with a focus on early-life immune development^[1]^. During pregnancy, there is a bias towards the production of T helper (Th) 2 cells at the maternal-fetal interface that induces maternal tolerance of the fetus^[2,3]^. Following birth, the development of the Th1 immune response in infants, believed to be mitigated by exposure to environmental microbial components, resets the Th1/Th2 balance^[4]^. However, low levels of circulating Th1-associated cytokines and high levels of Th2-associated cytokines in infants have been linked with sensitization and allergic diseases^[5]^. Thus, early-life microbiota exposure may provide opportunities to prevent allergy risks caused by the imbalance of Th1/Th2^[6]^.

*B. longum* subsp. *infantis* is an important bacterium in infant gut^[7]^ and is associated with several beneficial effects such as the production of bioactive substances, immature immune system maturation, and improvement of intestinal barrier integrity^[8,9]^. One of the factors that helps *B. longum* subsp. *infantis* to colonize the infant intestine is its capacity to digest the oligosaccharides found in human milk^[7,10]^. Studies have reported that loss of *Bifidobacterium* in early life is related to a high risk of several immune diseases and enteric inflammation, but the mechanism is unclear^[11,12]^. A recent publication has reported that *Lactobacillus casei* NCU011054 could relieve intestinal immune dysfunction induced by cyclophosphamide in immunosuppressive mice through the TLR/NF-κB pathway and then regulated Th1/Th2 immune balance^[13]^; *Lactobacillus plantarum* NCU116 and *Lactobacillus plantarum* 19-2 may promote Th1 immune response, thereby regulating intestinal immune in mice with cyclophosphamide-induced intestinal injury^[14,15]^; *Lactobacillus casei* LCR35 inhibits the occurrence of atopic dermatitis by regulating Th1/Th2 balance and gut microbiota^[16]^; Supplementing probiotics during weaning also contributes to the immune balance of infants. For example, feeding *Lactobacillus paracasei* F19 can reduce the cumulative incidence rate of eczema^[17]^, prevent the early manifestations of allergy, and enhance the immune response mediated by T cells^[18]^; *Lactobacillus rhamnosus* reduces the incidence rate of eczema in children^[19]^. Intestinal Th2 and Th17 cytokines decreased in breastfed infants after being administered with *B. longum* subsp. *infantis*^[20]^. Administration of EVC001 to adult mice had a therapeutic effect on allergic asthma by accelerating Th1 and silencing Th2 immune responses^[21]^. Additionally, *B. longum* subsp. *infantis* EVC001 led to the maintenance of the intestinal microenvironment^[11]^ by remodeling the intestinal microbiome in breastfed infants^[22]^. However, it is unclear if there are strain-specific variations among members of the *B. longum* subsp. *infantis* taxon in terms of their ability to regulate the Th1/Th2 immune balance following infant supplementation. Furthermore, gender differences impact the immune system in a microbiota-independent manner that selects for a gender-specific microbiota that further contributes to gender differences in immunity^[23]^.

Therefore, it makes sense to assess various *B. infantis* strains for their ability to regulate the Th1/Th2 balance and evaluate the function in both female and male mice. This study screens the effect of eight *B. longum* subsp. *infantis* strains on mice isolated in our laboratory previously. Its effect on the abundance of the major innate, adaptive immune cells, the differentiation markers of Th1/Th2 were evaluated using immunofluorescence, flow cytometry, and ELISA test, as well as the relative expression of JAK/STAT pathway at both transcription and translation level and the gut microbiota.

## METHOD AND MATERIALS

### Animal experiments

The ethical number of animal study was JN.No20210915b0601125[299]. Six-week-old females and males were specific-pathogen-free (SPF) BALB/c mice. Female (weight, 18 ± 1 g) and male (weight, 20 ± 1 g) mice (2:1) were kept in polypropylene cages at 20-26 °C and 40% to 70% humidity after one week of adaptation with a light-to-dark cycle of 12:12 h under hygienic conditions. The mice were fed with Co-60 gamma irradiation experimental mouse feed. Male mice were taken out after confirming that female mice were pregnant. The pregnant mice did not receive any treatment before delivery. Both males and females were gavaged with normal saline and different *Bifidobacterium longum* subsp. *infantis* strains I2MI (FJSWXI2MIM1), I4MI [FJSWXI4MI (CCFM1270)], I4MNI (FJSWXI4MNIM1), I5TI (FJSWXI5TIM1), I6TI (FJSWXI6TIM1), I8TI [FJSWXI8TI (CCFM1271)], I10TI [FJSWXI10TI (CCFM1272)], B6MNI [BJSWXB6MNIM1 (CCFM1269)], *n* = 8 per group, the details of *B. longum* subsp. *infantis* can be found in Supplementary Materials, respectively, from 1-week-old to 3-week-old with 1 × 10^9^ CFU/day/mice [Supplementary Figure 1]. *B. longum* subsp. *infantis* were adjusted to a uniform concentration after culture and kept in a 30% sucrose solution. Additionally, the *B. longum* subsp. *infantis* groups were kept in an identical amount of 30% sucrose solution at -80 °C, serving as the control group. The 30% sucrose solution in *B. longum* subsp. *infantis* groups and control group was extracted by centrifugation, rinsed three times in saline (0.9% NaCl), and then resuspended in saline before gavage. To counteract the sucrose impact, the control group received the same care as the *B. longum* subsp. *infantis* group. The same strain intervention group included all the pups from the same maternal mice. If there were less than eight puppies in each litter for females and males, the pups were combined. Even though they were grouped, maternal mice continued to feed their pups from separate litters. All the 3-week-old mice were sacrificed. Feces were collected before being sacrificed and stored at -80 °C with 30% glycerin. A blood sample (100 μL), mesenteric lymph nodes (MLNs), and colon were collected. Part of the colon was fixed in 4% formalin for immunofluorescence (IF). MLNs were used for flow cytometry (FCM). The materials and reagents used are listed in the Supplementary Materials.

### Flow cytometry and immunofluorescence analysis

MLNs were pulverized and passed through a 200-mesh cell sieve before adding an FC block agent (Miltenyi Biotec, Bergisch Gladbach, Germany). Cells were collected and antibodies of CD3, CD4, and CD19 (Abcam, Cambridge, UK) were added according to the instructions. The tube was incubated at 4 °C in the dark for 30 min, then supplemented with flow staining buffer, and centrifuged at 4 °C, 400 *g* for 5 min. The sediment was mixed with flow staining buffer before being analyzed using flow cytometry.

The colon tissues were fixed in 4% paraformaldehyde and subjected to IF test. The slices were dehydrated through gradient ethanol and then washed twice with PBS. Primary and secondary antibody staining were performed on the slices after the antigen was repaired. The slices were sealed with anti-fluorescence quenching agents after being washed with PBS three times. IF was automatically scanned with a 3D HISTECH, Budapest, Hungary, Panoramic MIDI device.

### Biochemical indicators measurement

Following a 10-minute centrifugation of whole blood at 3,500 *g*, serum samples were obtained. Subsequently, 50 mg colon was homogenized with PBS and the supernatant was collected for further analysis. The levels of IL-4, IFN-γ, IgE, and IgG2a in the serum and colon were assayed using ELISA kits following the instructions [Supplementary Materials].

### Quantitative real-time polymerase chain reaction analysis

Total RNA from PPs, colon, jejunum, and ileum tissue was extracted. Nanodrop was used to measure the RNA concentration. Quantitative real-time polymerase chain reaction (qRT-PCR) was utilized for amplification with SYBR green, and computed using the 2^-ΔΔCt^ technique, with β-actin serving as the internal standard. The primer sequences were searched on NCBI and primer bank, which are listed in Supplementary Table 1. qPCR conditions can be found in Supplementary Materials.

### Analysis of *16S rRNA* gene and Bifidobacterial *groEL* gene sequencing

The microbiota composition of fecal samples was sequenced by 16S rRNA V3-V4 region and the bifidobacterial *groEL* gene, following established protocols^[24]^. The amplified products were sequenced by Illumina MiSeq (San Diego, CA, US) and raw data were filtered by DADA2 and analyzed by Qiime 2^[25]^. PCR primers and conditions can be found in Supplementary Materials.

### Western blot analysis

The Western blot procedure was carried out following a previously published method^[26]^. The relative expression of target proteins was quantified by β-actin with ImageJ software^[27]^.

### Statistical analyses

The results were performed as the mean ± standard error of the mean (SEM). Data of FCM was analyzed by FlowJ (V10.06)^[28]^. One-way ANOVA was used to compare the difference among groups of more than two when the data met the criterion of normal distribution. Post hoc Tukey’s test was used to assess any significant differences between groups. “Table.Qzv” provided the sample resampling depth [Supplementary Materials], which is typically the minimal sample data amount or the data amount encompassing the great majority of samples. QIIME2 computed this depth. Alpha diversity was analyzed in QIIME2. Beta diversity was analyzed using PCoA (Principal Co-ordinates Analysis) based on the Bray-Curtis matrix conducted using the R package (“vagan”, “ape” and “ggplot2”).

## RESULTS

### Effects of *B. longum* subsp. *infantis* on immune cells of mice

**Figure 1:**
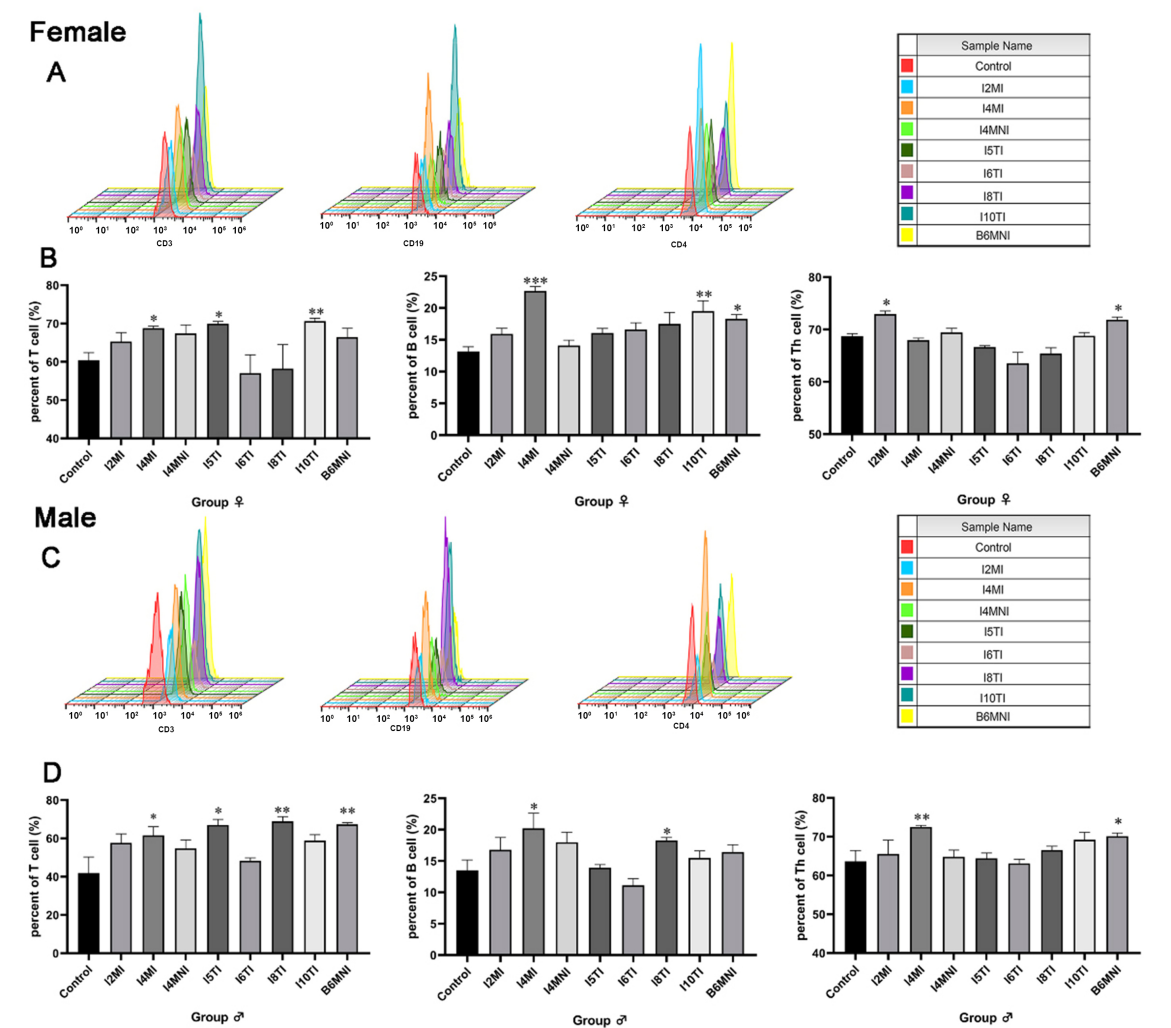
Effect of *B. longum* subsp. *infantis* on the percentage of B, T, and Th cells in mesenteric lymph nodes of female and male mice. (A and C) the positive peak of T cells (CD3 marker); B cells (CD19 marker), and Th cells (CD4 marker) of female and male mice, respectively; (B and D) Bar plot of percentage of B, T, and Th cells of female and male mice, respectively. ^*^P < 0.05; ^**^P < 0.01; ^***^P < 0.001, compared to corresponding gender control group. The mean ± SEM (*n* = 8 per group) was used to represent the data. Th: T helper; SEM: error of the mean.

IF and flow cytometry were used to assess the effects of *B. longum* subsp. *infantis* on the innate immune (CD11c-positive cells and macrophages, Supplementary Figures 2A and 3A) and adaptive immune cells (T cells, B cells, and Th cells, Supplementary Figures 4 and 5). The results revealed that *B. longum* subsp. *infantis* I4MI and B6MNI significantly increased the relative number of macrophages in female mice (*P* < 0.05, Supplementary Figure 2B). I2MI, I10TI, and B6MNI significantly increased the relative number of CD11c-positive cells in female mice (*P* < 0.05, Supplementary Figure 2C), and I2MI, I4MI, I8TI, and B6MNI significantly increased the relative number of macrophages in male mice (*P* < 0.05, Supplementary Figure 3B). I2MI, I4MI, I5TI, I8TI, and B6MNI significantly increased the relative number of CD11c-positive cells in male mice (*P* < 0.05, Supplementary Figure 3C). The positive peaks of T cells, B cells, and Th cells in female and male mice were analyzed. For female mice [Figure 1A], *B. longum* subsp. *infantis* I4MI, I5TI, and I10TI significantly increased the percentage of T cells (*P* < 0.05), I4MI, I10TI, and B6MNI significantly increased the percentage of B cells (*P* < 0.05, respectively), and I2MI and B6MNI increased the percentage of Th cells significantly (*P* < 0.05 for all, Figure 1B). For male mice [Figure 1C], *B. longum* subsp. *infantis* I4MI, I5TI, I8TI, and B6MNI significantly increased the percentage of T cells (*P* < 0.05), I4MI and I8TI significantly increased the percentage of B cells (*P* < 0.05), and I4MI and B6MNI significantly increased the percentage of Th cells (*P* < 0.05, respectively, Figure 1D).

### Effects of *B. longum* subsp. *infantis* on cytokines and immunoglobulins in the colon of mice

**Figure 2:**
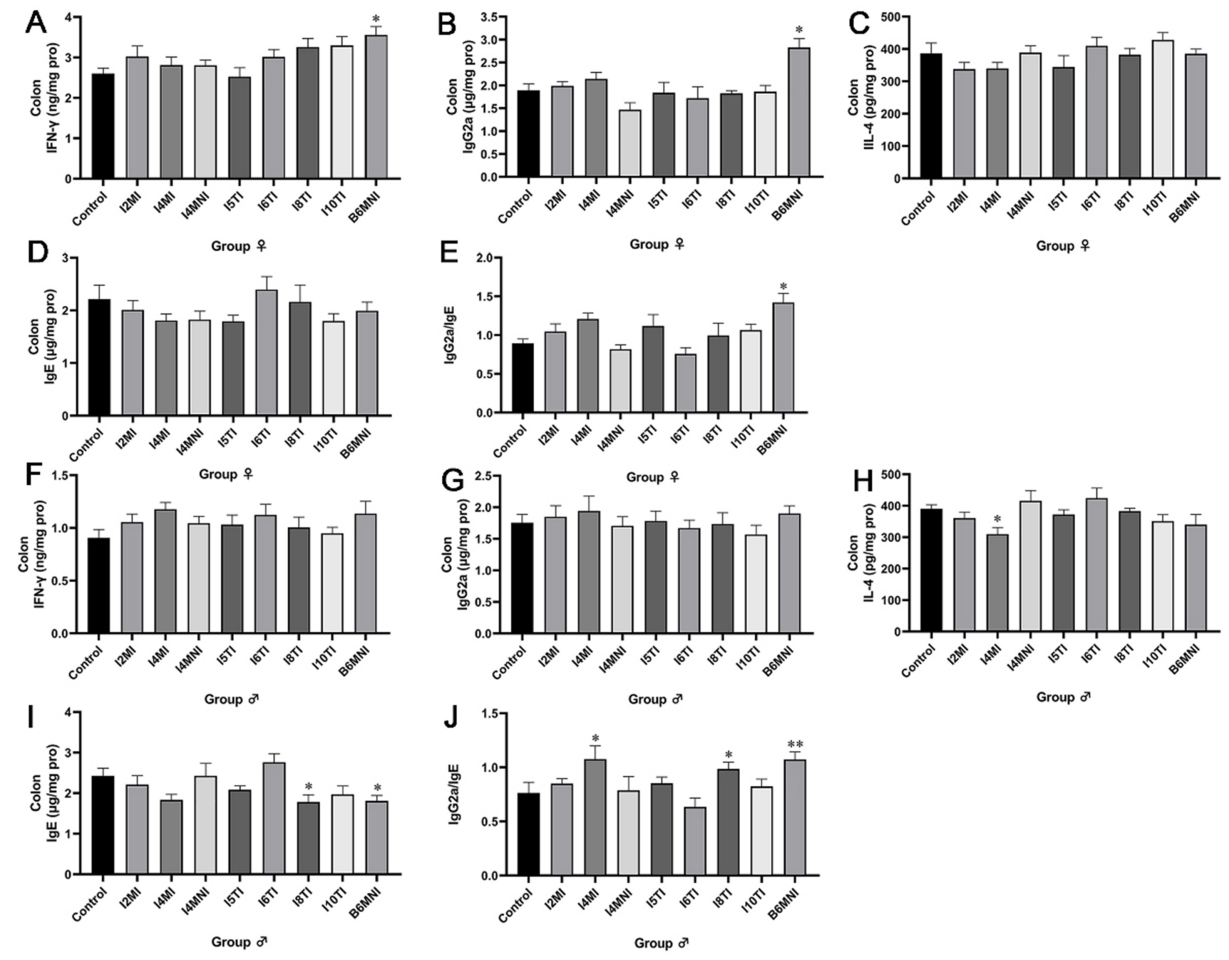
Effects of *B. longum* subsp. *infantis* on the levels of cytokines and Immunoglobulin in the colon of neonatal female (A-E) and male mice (F-J). (A) IFN-γ; (B) IgG2a; (C) IL-4; (D) IgE; (E) the ratio of IgG2a and IgE; (F) IFN-γ; (G) IgG2a; (H) IL-4; (I) IgE; (J) the ratio of IgG2a and IgE. ^*^P < 0.05, ^**^P < 0.01, compared to corresponding gender control group. The mean ± SEM (*n* = 8 per group) was used to represent the data. SEM: Error of the mean.

The ability of eight *B. longum* subsp*. infantis* strains to stimulate immune cells capable of producing Th1/Th2-associated gene proteins was first assessed by immunofluorescence and flow cytometry. The balance of Th1/Th2 was assessed by cytokines and immunoglobulins. *B. longum* subsp. *infantis* B6MNI increased the level of IFN-γ and IgG2a significantly (*P* < 0.05, Figure 2A and B) in the colon of female mice. No significant differences were found in the levels of IL-4 and IgE in the colon of female mice [Figure 2C and D]. B6MNI increased the ratio of IgG2a/IgE (*P* < 0.05, Figure 2E) in the colon of female mice. For male mice, no significant differences were found in the levels of IFN-γ and IgG2a in the colon for female mice [Figure 2F and G]. *B. longum* subsp. *infantis* I4MI decreased the level of IL-4 (*P* < 0.05, Figure 2H), while I8TI and B6MNI decreased the level of IgE (*P* < 0.05, Figure 2I) significantly. Furthermore, I4MI, I8TI, and B6MNI increased the ratio of IgG2a/IgE significantly (*P* < 0.05, Figure 2J).

### Effects of *B. longum* subsp. *infantis* on mRNA relative expression of JAK/STAT pathway in the colon of mice

**Figure 3:**
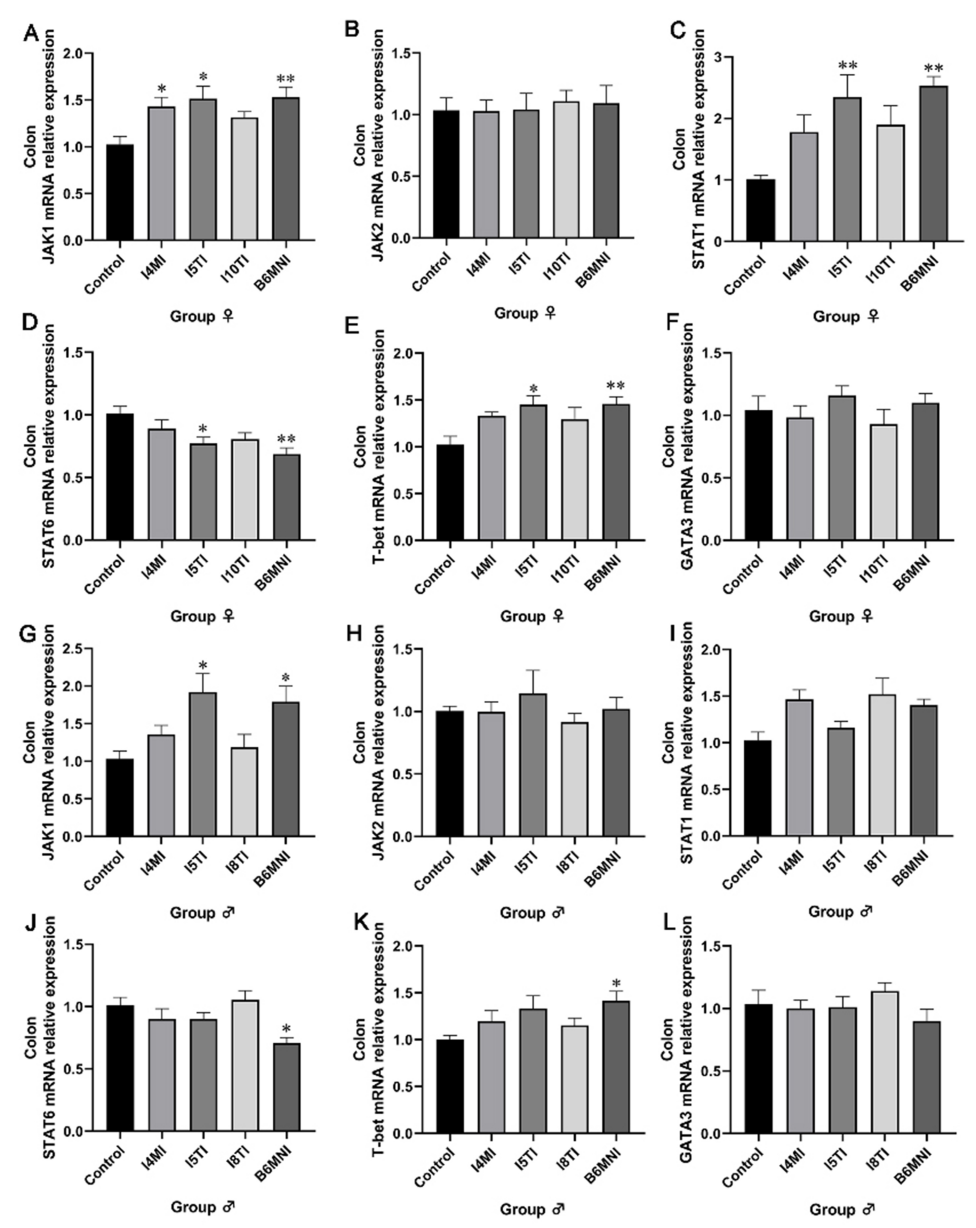
Effects of *B. longum* subsp. *infantis* on the expression of the related genes in the JAK/STAT pathway in the colon of female (A-F) and male mice (G-L). (A) JAK1; (B) JAK2; (C) STAT1; (D) STAT6; (E) T-bet; (F) GATA3; (G) JAK1; (H) JAK2; (I) STAT1; (J) STAT6; (K) T-bet; (L) GATA3. ^*^P < 0.05, ^**^P < 0.01, compared to corresponding gender control group. The mean ± SEM (*n* = 8 per group) was used to represent the data. SEM: Error of the mean.

Based on the described results, *B. longum* subsp. *infantis* I4MI, I5MI, I10TI, and B6MNI for female mice, and I4MI, I5TI, I8TI, and B6MNI for male mice can adjust Th1/Th2 related genes and proteins. Therefore, these strains were selected for subsequent analysis. The expression of genes associated with the JAK/STAT pathway in the colon was assessed by qRT-PCR to analyze the effects of *B. longum subsp. infantis* on their transcriptional levels. The relative abundance of JAK1 mRNA in the I4MI, I5TI, and B6MNI groups was significantly higher than that in the female control group (*P* < 0.05, Figure 3A). No significant differences were found in the relative expression of JAK2 in the colon of female mice [Figure 3B]. The relative expression of STAT1 mRNA was significantly higher in I5TI (*P* < 0.05, respectively) and B6MNI groups (*P* < 0.01 for both, Figure 3C) and the relative expression of STAT6 mRNA was significantly lower in I5TI (*P* < 0.05) and B6MNI groups (*P* < 0.01, Figure 3D) compared to that in the female control group. The relative expression of T-bet mRNA was significantly higher in I5TI (*P* < 0.05, respectively) and B6MNI groups (*P* < 0.01 for both, Figure 3E). No significant differences were found in the relative expression of GATA3 in the colon of female mice [Figure 3F]. For male groups, *B. longum* subsp. *infantis* I5TI and B6MNI increased the JAK1 mRNA relative expression (*P* < 0.05, Figure 3G). No significant differences were found in the relative expression of JAK2 and STAT1 in the colon of male mice [Figure 3H and I], while B6MNI decreased the STAT6 mRNA relative expression (*P* < 0.05, Figure 3J) and increased the T-bet mRNA relative expression significantly (*P* < 0.05, Figure 3K). No significant differences were found in the relative expression of GATA3 in the colon of male mice [Figure 3L].

### Effects of *B. longum* subsp. *infantis* on the JAK/STAT pathway in the colon of mice

**Figure 4:**
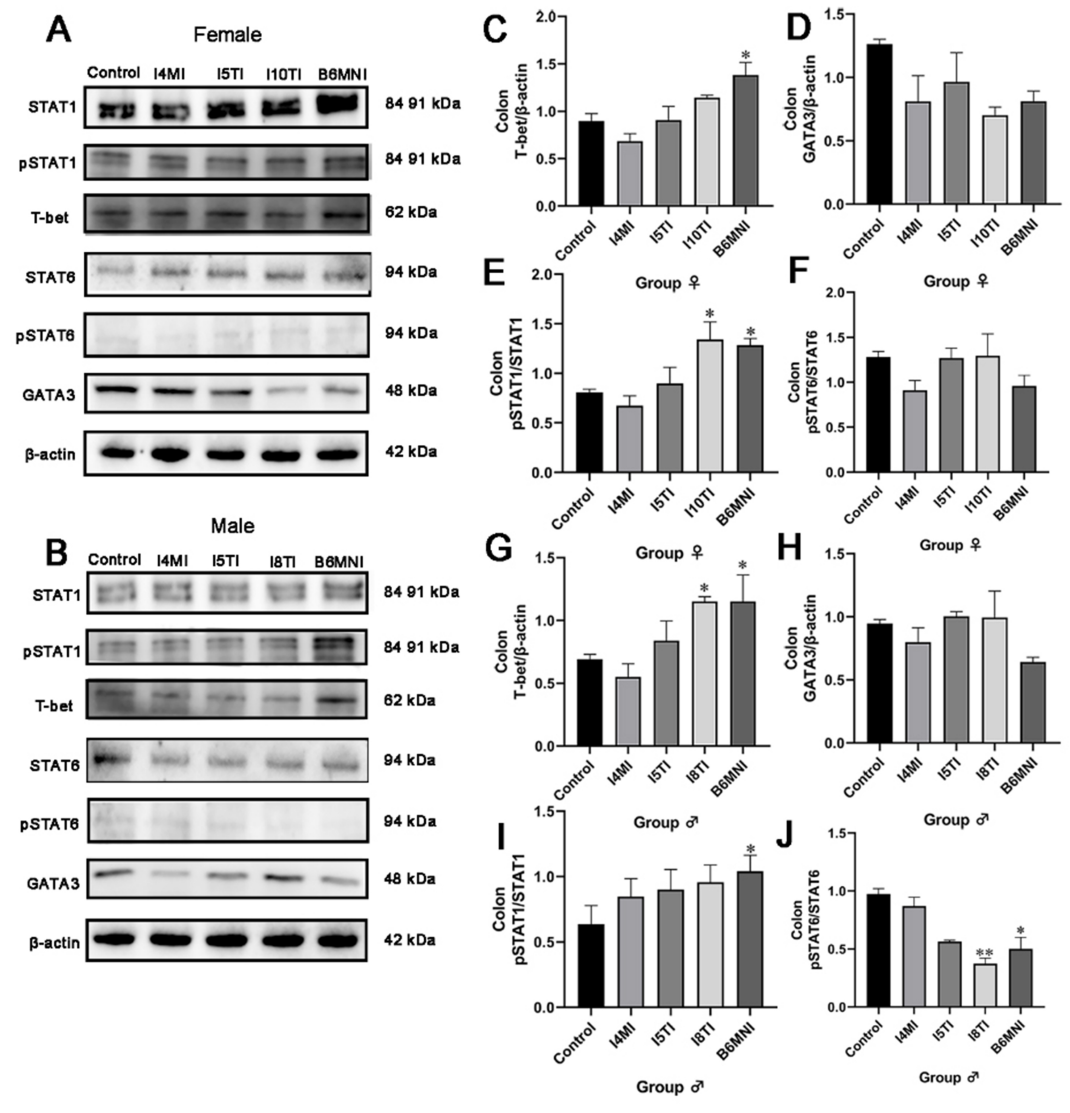
Effects of *B. longum* subsp. *infantis* on JAK/STAT pathway-related protein expression in the colon of female mice (C-F) and male mice (G-J). (A) colon STAT1, pSTAT1, T-bet, STAT6, pSTAT6, GATA3 western blotting bands of female mice; (B) colon STAT1, pSTAT1, T-bet, STAT6, pSTAT6, GATA3 western blotting bands of male mice; (C) T-bet; (D) GATA3; (E) pSTAT1; (F) pSTAT6; (G) T-bet; (H) GATA3; (I) pSTAT1; (J) pSTAT6. ^*^P < 0.05, compared to the corresponding gender control group. The mean ± SEM (*n* = 8 per group) was used to represent the data. SEM: Error of the mean.

The translational level of corresponding genes was verified through western blot analysis [Figure 4A and B]. The relative expression of T-bet was significantly higher only in the female mice treated with B6MNI compared with the control group (*P* < 0.05, Figure 4C). No significant differences were found in the relative expression of pSTAT6 for female mice [Figure 4D]. The relative expression of pSTAT1 was significantly higher in the female mice fed with I10TI and B6MNI compared with the control group (*P* < 0.05 for all, Figure 4E). No significant differences were found in the relative expression of GATA3 for female mice [Figure 4F]. Additionally, *B. longum* subsp. *infantis* B6MNI and I8TI increased the relative expression of T-bet (*P* < 0.05, Figure 4G). No significant differences were found in the relative expression of GATA3 in colon tissues from either female or male mice [Figure 4H]. *B. longum* subsp. *infantis* B6MNI increased the relative expression of pSTAT1 (*P* < 0.05, Figure 4I), while it decreased the relative expression of pSTAT6 (*P* < 0.05, Figure 4J) in male mice compared with the control group. *B. longum* subsp. *infantis* I8TI decreased the level of pSTAT6 (*P* < 0.01, Figure 4J) in male mice compared with the control group.

### Effects of *B. longum* subsp. *infantis* on the gut microbiota in mice

Total gut microbiota was analyzed by *16S rRNA* gene sequencing. The mice of different genders showed different results. For female mice, Chao 1 and Shannon indexes showed no significant differences among groups for total bacteria [Supplementary Figure 6A and B]. For male mice, the Chao 1 index for total bacteria was significantly lower in I4MI (*P* < 0.05), I5TI (*P* < 0.01), and I8TI (*P* < 0.01) groups compared with that in the control [Supplementary Figure 6C]. No significant was found of Shannon index in male mice [Supplementary Figure 6D]. PCoA revealed significantly different bacterial compositions for total bacteria among the five groups (*P* = 0.001, Supplementary Figure 6E and F). The top 30 total bacteria were presented in Supplementary Figure 6G. For total bacteria, the dominant member in the control group was *Lactobacillus* (25.23%), whereas unclassified *Muribaculaceae* (19.07%), *Bacteroides* (14.39%), *Enterococcus* (25.18%), and *Alistipes* (15.69%) became dominant in I4MI, I5TI, I10TI, and B6MNI treated groups, respectively [Supplementary Figure 6G]. *Lactobacillus* and unclassified *Muribaculaceae* were dominant in the control (15.98%, 15.44%) and I8TI groups (17.71%, 18.19%), *Bacteroides* (18.61%) and *Lactobacillus* (17.34%) were predominant in the I4MI group, the I5TI group was dominated by *Bacteroides* (26.51%), while the B6MNI group was dominated by *Enterococcus* (20.47%, Supplementary Figure 6H). The relative abundance of *Bifidobacterium* in I4MI, I5TI, and B6MNI groups was significantly higher than that in the control (*P* < 0.05, Supplementary Figure 6I), and the relative abundance of *Enterococcus* was significantly higher in I10TI (*P* < 0.001) and B6MNI (*P* < 0.001, Supplementary Figure 6J) treated mice. Similar to that in female mice, *B. longum* subsp. *infantis* I4MI, I5TI, I8TI, and B6MNI treatments increased the relative abundance of total *Bifidobacterium* (*P* < 0.05, Supplementary Figure 6K), whereas the relative abundance of *Enterococcus* was lower in the I8TI-treated mice but higher in the B6MNI group compared with that in the control group (*P* < 0.05, Supplementary Figure 6L).

**Figure 5:**
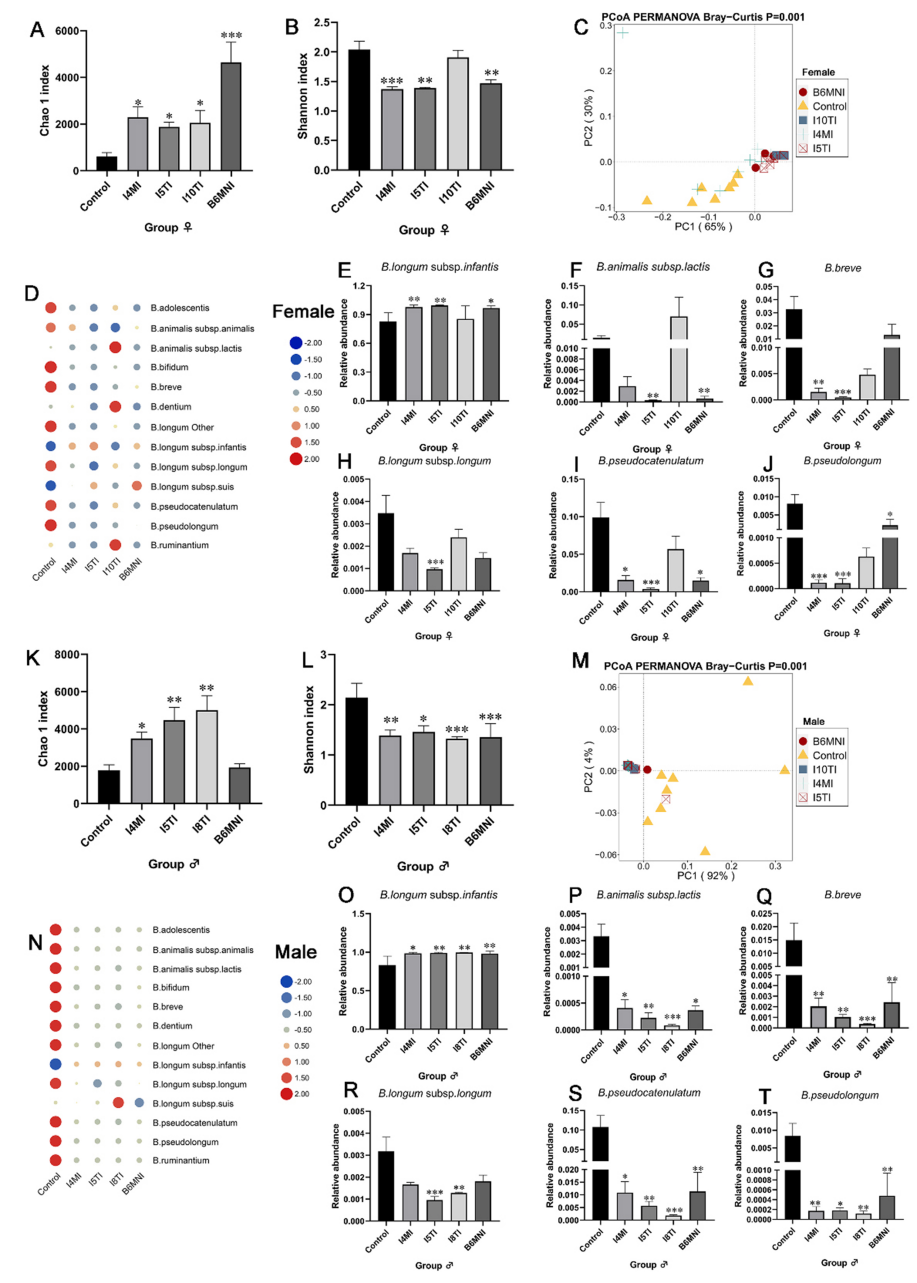
Effects of *B. longum* subsp. *infantis* on the diversity of the bifidobacterial community in female and male mice. (A and B) Chao 1 and Shannon index of total bacteria in female mice; (C) β-diversity of *Bifidobacterium* composition in female mice; (D) heatmap of bifidobacterial composition in female mice. The relative abundance of *Bifidobacterium* in each group was row normalization. The blue circle means lower relative abundance and the red circle means higher relative abundance; (E-J) the significant difference of *Bifidobacterium* in female mice; (K and L) Chao 1 and Shannon index of total bacteria in male mice; (M) β-diversity of bifidobacterial community in male mice, respectively; (N) heatmap of bifidobacterial composition in male mice. The relative abundance of *Bifidobacterium* in each group was row normalization. The blue circle means lower relative abundance and the red circle means higher relative abundance; (O-T) the significant difference of *Bifidobacterium* in male mice. ^*^P < 0.05, ^**^P < 0.01, ^***^P < 0.001, compared to corresponding to the control group. The mean ± SEM (*n* = 8 per group) was used to represent the data. SEM: Error of the mean.

The bifidobacterial community in each group was analyzed by bifidobacterial *groEL* gene sequencing. For female mice, I4MI, I5TI, I8TI, and B6MNI increased the Chao 1 index (*P* < 0.05), but I4MI, I5TI, and B6MNI decreased the Shannon index (*P* < 0.01, Figure 5A and B). PCoA results revealed that the *Bifidobacterium* composition in the five groups was significantly different (*P* = 0.001, Figure 5C). *B. longum* subsp. *infantis* was dominant in the control (82.84%), I4MI (97.66%), I5TI (99.40%), I8TI (85.40%), and B6MNI groups (96.70%, Figure 5D). Furthermore, the relative abundance of *B. longum* subsp. *infantis* was significantly higher in I4MI, I5TI, and B6MNI groups than that in the control group (*P* < 0.05, Figure 5E). In contrast, the relative abundance of *B. animalis* subsp. *lactis* was significantly lower in I5MI (*P* < 0.01) and B6MNI groups (*P* < 0.05) compared to that in the control group [Figure 5F]. The relative abundance of *B. breve* was significantly lower in I5MI (*P* < 0.01) and I4MI groups (*P* < 0.05) compared to that in the control group [Figure 5G]. The relative abundance of *B. longum* subsp. *longum* was significantly lower in I5MI (*P* < 0.001) compared to that in the control group [Figure 5H]. In contrast, the relative abundance of *B. pseudocatenalatum*, and *B. pseudolongum* were significantly lower in I4MI, I5MI, and B6MNI groups compared to that in the control group (*P* < 0.05, Figure 5I and J).

Similar to the effects of *B. longum* subsp. *infantis* on the bifidobacterial community in female mice, the Chao 1 index for male mice was significantly higher in I4MI, I5TI, and I8TI groups, while the Shannon index was significantly lower in the I4MI, I5TI, I8TI, and B6MNI groups (*P* < 0.05, Figure 5K and L). The bifidobacterial profile was significantly different among the five groups (*P* = 0.001, Figure 5M). *B. longum* subsp. *infantis* was the predominant *Bifidobacterium* species in the control (83.37%), I4MI (98.45%), I5TI (99.14%), I8TI (99.60%), and B6MNI groups (98.21%, Figure 5N). The relative abundance of *B. longum* subsp. *infantis* was significantly higher, but the relative abundances of *B. animalis* subsp. *lactis*, *B. breve*, *B. pseudocatenalatum*, and *B. pseudolongum* were significantly lower in I4MI, I5TI, I8TI, and B6MNI groups compared to that in the control group (*P* < 0.05, Figure 5O-T). Moreover, I5TI and I8TI treatments showed a lower relative abundance of *B. longum* subsp. *longum* (*P* < 0.01, Figure 5R).

### Effects of *B. longum* subsp. *infantis* on cytokines and immunoglobulins in serum

**Figure 6:**
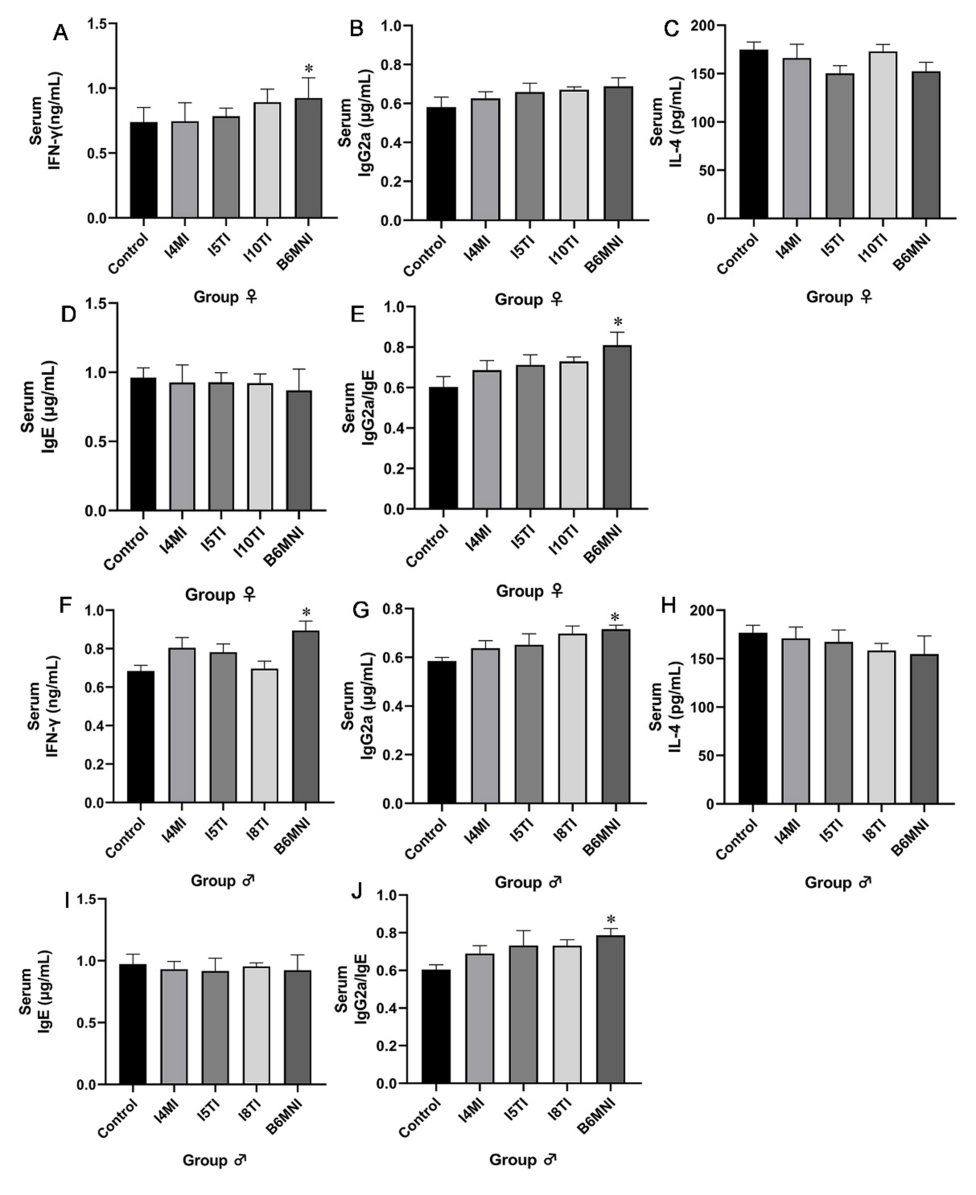
Effects of *B. longum* subsp. *infantis* on the level of cytokines and immunoglobulin in serum in neonatal female (A-E) and male mice (F-J). (A) IFN-γ; (B) IgG2a; (C) IL-4; (D) IgE; (E) the ratio of IgG2a and IgE; (F) IFN-γ; (G) IgG2a; (H) IL-4; (I) IgE; (J) the ratio of IgG2a and IgE. ^*^P < 0.05, compared to the corresponding gender control group. The mean ± SEM (*n* = 8 per group) was used to represent the data. SEM: Error of the mean.

Th1/Th2-related cytokines in the serum were further determined to assess the regulation of strains on the balance of Th1/Th2 in peripheral blood of the organism. Only B6MNI increased the level of IFN-γ (*P* < 0.05, Figure 6A) in female mice. Four strains had no significant effects on the level of IgG2a, IL4, and IgE [Figure 6B-D]. B6MNI increased the ratio of IgG2a/IgE (*P* < 0.05, Figure 6E) in female mice. In the males, B6MNI increased the level of IFN-γ (*P* < 0.05, Figure 6F) and IgG2a (*P* < 0.05, Figure 6G). Four strains had no significant effects on the level of IL4 and IgE [Figure 6H and I]. B6MNI increased the IgG2a/IgE ratio (*P* < 0.05, Figure 6J).

## DISCUSSION

In this study, we evaluated the inflammatory cytokines, genes, and proteins related to the JAK/STAT signaling pathway that regulate the Th1/Th2 immune balance and gut microbiota in mice from 1 week-old to 3-week-old that had been gavaged with eight different *Bifidobacterium longum* subsp. *infantis* strains (I2MI, I4MI, I4MNI, I5TI, I6TI, I8TI, I10TI, and B6MNI). Our findings indicated that eight strains *B. longum* subsp. *infantis* may adjust the Th1/Th2 balance in the colon and serum in female and male mice but with strain specificity, among which *B. longum* subsp. *infantis* B6MNI was more pronounced compared to the other seven strains.

The Th1/Th2 balance is related to the occurrence and development of allergy, asthma, and eczema^[29]^. *B. longum* subsp. *infantis*, an important microorganism in infant gut, may adjust the Th1/Th2 immune balance in both male and female mice in this study. IFN-γ and IL-4 are the cytokines released by Th1 and Th2 cells, respectively. Specifically, *B. longum* subsp. *infantis* B6MNI was shown to increase the level of IFN-γ significantly in female mice but failed to increase this in male mice. Additionally, *B. longum* subsp. *infantis* B6MNI also stimulated B cells to release Th1 type immunoglobulin IgG2a and increased the ratio of IgG2a/IgE, which refers to the Th2 immune type transferring to the Th1 immune type. However, in males, *B. longum* subsp. *infantis* B6MNI failed to increase the cytokines and immunoglobulin related to the Th1 immune type but decreased the IgE related to the Th2 immune type. Hence, we proposed that the regulatory effect of *B. longum* subsp. *infantis* B6MNI on female mice was mainly based on promoting the secretion of Th1 cytokines, while its effect on male mice was mainly based on inhibiting the secretion of Th2 cytokines, which may be influenced by gender^[30]^. Currently, it has been shown that *Bifidobacterium* could enhance the intestinal immune barrier, but their regulation of serum indicators is limited^[31]^. The establishment of Th1/Th2 immune balance can prevent the occurrence and address the development of allergic diseases. Hence, administration of *Bifidobacterium* in infants may help prevent and alleviate allergic diseases. Intervention with specific strains of mice after birth can promote Th1/Th2 immune balance, which may effectively prevent the incidence of allergies, asthma, and other diseases in childhood. A similar publication reported that administration of *Lactobacillus paracasei* in infants between 4 and 13 months of age can effectively reduce the incidence of allergies and asthma at the age of 8-9 by 11% and 3%, respectively^[17]^. Therefore, supplementation with specific probiotics in infants after birth has the potential to reduce the incidence of childhood diseases effectively.

JAK-STAT mediates the responses to cytokines and growth factors and is part of homeostasis and developmental processes^[32]^. T-bet and GATA3 are two important transcriptional factors for secreting IFN-γ and IL-4, respectively^[33]^. Deficiency of STAT1 and STAT6 leads to failure of releasing mature IFN-γ and IL-4^[34]^. *B. longum* subsp. *infantis* can release IFN-γ and inhibit the production of IL-4 by activating the JAK/STAT pathway but in a strain-specific manner. Interestingly, *B. longum* subsp. *infantis* I5TI increased the gene levels of the JAK/STAT pathway but failed to influence the level of cytokines and immunoglobulin related to Th1/Th2 in both male and female mice. The reason for this may be that its effect on these genes did not reach the level of translation, which was confirmed by western blot analysis in this study. *B. longum* subsp. *infantis* B6MNI not only significantly increased the gene expression level of the JAK/STAT pathway, but also increased their protein level in the colon. Strain specificity is exhibited in regulating both Th1/Th2-related gene proteins among *B. longum* subsp. *infantis*. The reason for the variability of those strains may be the characteristics of the strains themselves, for instance, different carbohydrate utilization^[35]^, and extracellular polysaccharide biosynthesis^[36]^. In addition, different metabolites of the strains *in vivo* or *in vitro* may also contribute to the variability of the strains^[37]^. Therefore, the reason for strain specificity needs further exploration.

*B. longum* subsp. *infantis* influenced gut microbiota composition in the mice. In this study, the variety of gut microbial composition was more pronounced in male mice compared to that in female mice. A previous study reported female mice with a higher relative abundance of *Bifidobacterium*^[38]^, which was inconsistent with our study. This gender-specific difference in intestinal microbiota may be related to estrogen^[39]^. The effect of *B. longum* subsp. *infantis* on the gut microbiota is reflected not only in the relative abundance of some genera but also in the presence or absence of a specific genus. For example, the relative abundance of *B. longum* subsp. *infantis* in the mice intestine was significantly increased after *B. longum* subsp. *infantis* intervention. The relative abundance of *B. longum* subsp. *infantis* in the infant gut is inversely associated with immune-mediated disease incidence during growth, which suggests the critical role of *B. longum* subsp. *infantis* during early-life immune system imprinting^[40]^. Therefore, these results indicate that the microbiota colonization that occurs in early life influences a pivotal window of immunological development.

*B. longum* can enhance the immune response of Th1 cells by stimulating immune cells to produce IFN-γ to promote the development and function of Th1 cells^[41]^. *B*. *longum* can also inhibit the activation and proliferation of Th2 cells, reduce allergic reactions and humoral immune responses, regulate cytokine balance in immune responses, such as increasing IL-10 production, and inhibit the development and function of Th2 cells^[42]^. Research has shown that the *B. longum* subsp. *infantis* can treat allergic asthma by regulating Th1/Th2 balance, including elevating the level of Th1 related cytokine and decreasing the level of Th2 cytokine^[21]^. Supplementing specific *Bifidobacterium* or *Lactobacillus* in the early stages of life to prevent the occurrence and development of allergic diseases in infants and young children can contribute to their healthy development. For example, *Lactobacillus rhamnosus* HN001 supplementation can reduce the incidence of eczema in infants by 50%^[43]^. Additionally, taking HN001 from birth to 2 years old can prolong the preventive or relieving effect on eczema to 4 years old^[43]^. This study found that 8 strains of *B. longum* subsp. *infantis* have different regulatory abilities on Th1/Th2 immune balance through mice models. Among them, B6MNI was more pronounced. *B. longum* subsp. *infantis* B6MNI increased relative abundance of *B. longum* subsp. *infantis* and regulates Th1/Th2 balance by affecting the JAK/STAT signaling pathway in both male and female mice. The other seven strains of *B. longum* subsp. *infantis* affected changes in gut microbiota but did not regulate the JAK/STAT signaling pathway. This may indicate that changes in microbial communities were not sufficient to affect the JAK/STAT signaling pathway and the difference in metabolites among strains *in vivo* or *in vitro* may contribute to this result. Furthermore, it was found significant differences in metabolites between B6MNI and seven other strains *B. longum* subsp. *infantis in vitro* (data not shown). B6MNI affected the content of tryptophan metabolites through a mouse model^[44]^, which may be the reason for its different effects compared to the other seven strains *B. longum* subsp. *infantis*. Therefore, *B. longum* subsp. *infantis* has the potential to prevent allergies and asthma despite its strain-specific. These regulatory effects are expected to improve the immune function of infants and young children and prevent the occurrence of allergic diseases. Exploring the intricacies of gut microorganisms and their responsible mechanisms is crucial for advancing the development of microbial formulations tailored for infants at risk of microbiota imbalances. Such imbalances could compromise immune maturation and increase susceptibility to disease. It is essential to understand these processes to enable the supplementation of specific probiotics in infants as early as possible after birth, thereby promoting their overall health. The limitations and gaps in this study were the need for further analysis of strain differences at the genetic level.

In conclusion, The regulation of Th1/Th2 in 3-week-old mice by eight *Bifidobacterium longum* subsp. *infantis* strains exhibited strain-specificity, mainly reflected in their effects on specific immune cells, Th1/Th2 immune balance, and gut microbiota. Among these strains, B6MNI showed the most significant regulatory ability, possibly by regulating colon Th1/Th2 differentiation biomarkers through the JAK-STAT pathway, promoting serum Th1 cytokines and immunoglobulin levels, and regulating gut microbiota composition, especially increasing the relative abundance of *Bifidobacterium* and *B. longum* subsp. *infantis*.

## DECLARATIONS

### Availability of data and materials

Raw sequencing reads were accessible from the National Center for Biotechnology Information (NCBI) Sequence Read Archive (SRA) database under the accession number PRJNA884502.

### Copyright

© The Author(s) 2024.
