# Supplementary material for: Bifidobacterium longum subsp. infantis regulates Th1/Th2 balance through the JAK-STAT pathway in growing mice
Source: Microbiome Res Rep. 2024 Jan 19;3(2):16. doi: 10.20517/mrr.2023.64 (PMC11149089; doi:10.20517/mrr.2023.64)
Supplement: Supplementary file 1 [file mrr-3-2-16-SupplementaryMaterials.pdf]

Supplementary Materials

Bifidobacterium longum subsp. _infantis_ regulates Th1/Th2 balance through the JAK-STAT pathway in growing mice

Mengfan Ding[1,2], Bowen Li[1,2], Haiqin Chen[1,2], R. Paul Ross[3,4], Catherine Stanton[3,4,5], Shilong Jiang[6,7], Jianxin Zhao[1,2,8], Wei Chen[1,2,8], Bo Yang[1,2,3]

[1]State Key Laboratory of Food Science and Resources, Jiangnan University, Wuxi 214122, Jiangsu, China.

[2]School of Food Science and Technology, Jiangnan University, Wuxi 214122, Jiangsu, China.

[3]International Joint Research Center for Probiotics & Gut Health, Jiangnan University, Wuxi 214122, Jiangsu, China.

[4]APC Microbiome Ireland, University College Cork, Cork T12 R229, Ireland.

[5]Teagasc Food Research Centre, Moorepark, Co. Cork P61 C996, Ireland.

[6]Nutrition and Metabolism Research Division, Innovation Center, Heilongjiang Feihe Dairy Co., Ltd, Beijing 100015, China.

[7]PKUHSC-China Feihe Joint Research Institute of Nutrition and Healthy Lifespan Development, Beijing 100083, China.

[8]National Engineering Research Center for Functional Food, Jiangnan University, Wuxi 214122, Jiangsu, China.

Correspondence to: Prof. Bo Yang, School of Food Science and Technology, Jiangnan University, 1800 Lihu Avenue, Wuxi 214122, Jiangsu, China. E-mail: bo.yang@jiangnan.edu.cn

Material and reagents

Eight strains of *B. longum* subsp. *infantis* were deposited at the Culture Collection of Food Microbiology, Jiangnan University, Wuxi, China which isolated by our lab. Among eight strains, the patent number of I4MI, I8TI, I10TI, and B6MNI are CCFM 1270, CCFM 1271, CCFM 1272, and CCFM1269. CCFM1269 were isolated from human breastmilk and the other seven strains were isolated from infant feces. Antibodies (CD3, CD4, and CD19) for flow cytometry, CD11c, and F4/80 for IF and anti-IgA for MACS were purchased from Abcam (Cambridge, UK). Paraformaldehyde was purchased from Servicebio (Wuhan, China). Enzyme-linked immunosorbent assay (ELISA) kits for interferon-gamma (IFN-γ), interleukin-4 (IL-4), immune globulin 2a (IgG2a) and immune globulin E (IgE) were purchased from Sangon Biotech (Shanghai, China); The bicinchoninic acid (BCA) assay kit was purchased from Thermo Fisher Scientific (Waltham, MA, USA). FastDNA Spin Kit for Feces was purchased from MP Biomedicals (Irvine, CA, USA). DNA Gel/PCR Purification Miniprep kit was purchased from BIOMIGA (San Diego, CA, USA). Tissue total RNA isolation kit, HiScript III All-in-one RT SuperMix Perfect for qPCR, and ChamQ SYBR qPCR Master Mix were purchased from Vazyme (Nanjing, China). RIPA lysis buffer was purchased from Beyotime (Shanghai, China). 2× Laemmli Sample Buffer was purchased from Bio-rad (Hercules, CA, USA). Primary antibodies STAT1, pSTAT1, T-bet, STAT6, pSTAT6, and GATA3 were purchased from Abcam (Cambridge, UK); Secondary antibody was supplied by Abcam (Cambridge, UK).

qPCR condition for qRT-PCR

qRT-PCR condition was 95 ℃ 5 min, 95 ℃ 30 s, 60 ℃ 30 s, 72 ℃ 2 min, 35 cycles, and 72 ℃ 10 min.

PCR primer and condition for 16S rRNA gene sequencing and bifidobacterial sequencing

The V3-V4 regions of the *16S rRNA* gene was PCR-amplified using primers 341F (5'-CCTAYGGGRBGCASCAG-3') and 806R (5'-GGACTACNNGGGTATCTAAT-3'). The bifidobacterial *groEL* gene was amplified using the primers Bif-*groEL*-F (5'-TCCGATTACGAYCGYGAGAAGCT-3')/Bif-*groEL*-R (5'-CSG CYTCGGTSGTCAGGA-ACA-G-3'). The PCR condition was 95 ℃ 5 min, 95 ℃ 30 s, 52 ℃ 30 s, 72 ℃ 30 s, 30 cycles, 72 ℃ 10 min. PCR reaction system consisted of 2× Taq 25 μL, 10 μM forward and backward primer 1 μL, DNA 1 μL, ddH_2O 22 μL.

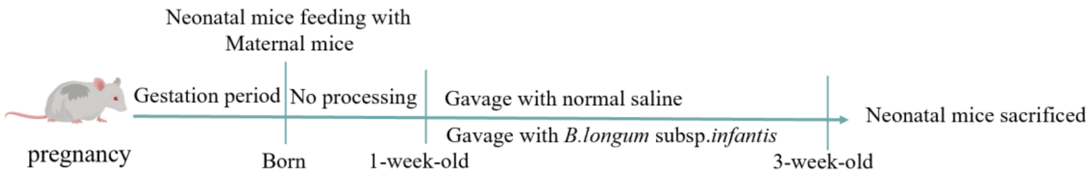

Supplementary Figure 1. Process of animal experiment. Pregnant maternal mice were housed in a single cage. After a 3-week gestation period, neonatal mice were born and housed with their mother until weaned at 3 weeks old. Neonatal mice were gavaged with normal saline (female and male group, n = 8 per group) and with eight strains of *B. longum* subsp. *infantis* from 1 week to 3 weeks old. And then the neonatal mice were sacrificed. The eight strains of *B. longum* subsp. *infantis* are I2MI, I4MI, I4MNI, I5TI, I6TI, I8TI, I10TI, and B6MNI, respectively.

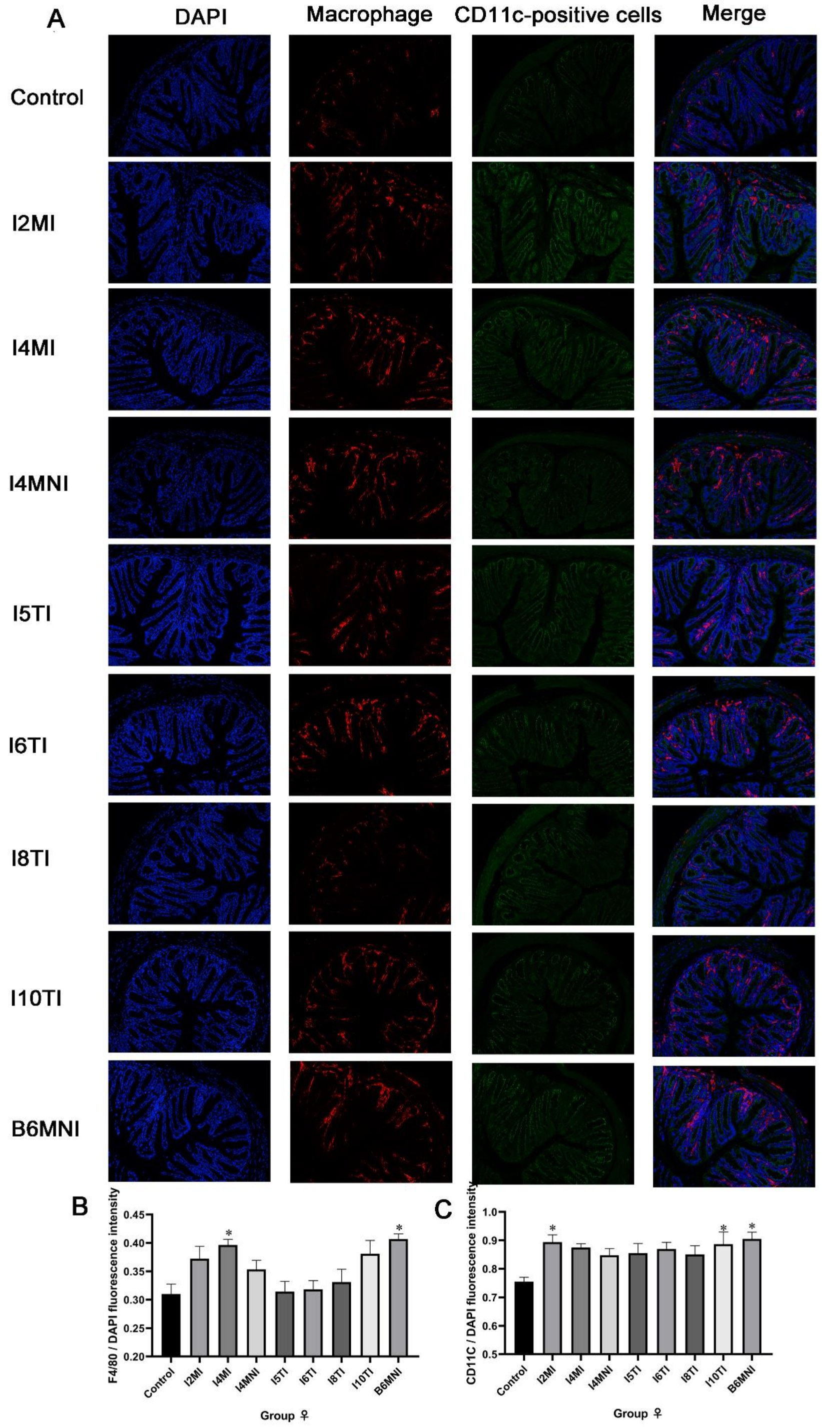

Supplementary Figure 2. Effect of *B. longum* subsp. *infantis* on the content of CD11c-positive cells and macrophage cells in the colon of female mice (n = 3 per group). Macrophages were marked by red fluorescence and CD11c-positive cells were marked by green fluorescence. The magnification is 200×. $^{*}P$<0.05, compared to the corresponding gender control group. (A) immunofluorescence of CD11c-positive cells and macrophage cells; (B) the relative number of macrophage cells; (C) the relative number of CD11c-positive cells.

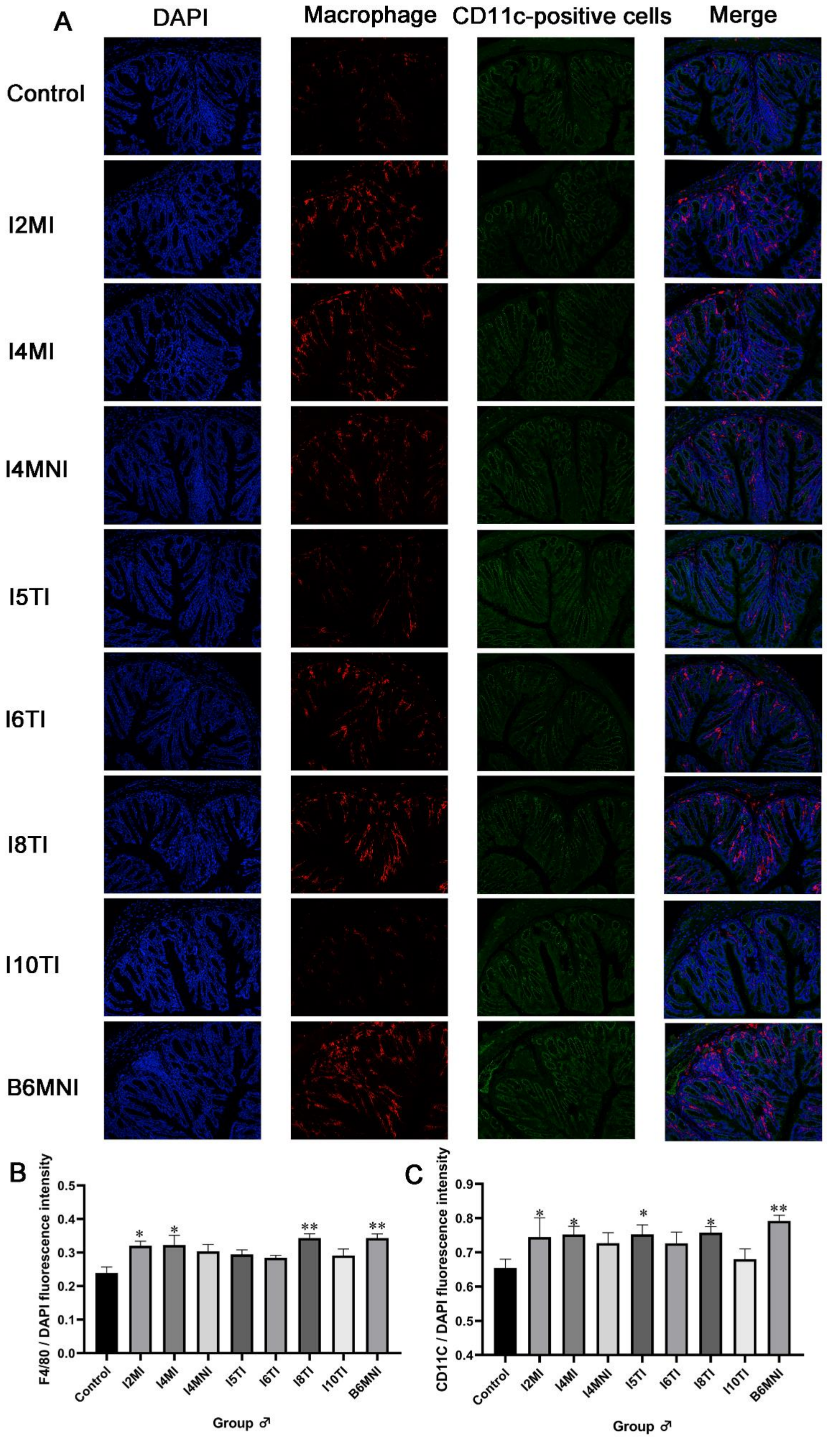

Supplementary Figure 3. Effect of *B. longum* subsp. *infantis* on the content of CD11c-positive cells and macrophage cells in the colon of male mice (n = 3 per group). Macrophages were marked by red fluorescence and CD11c-positive cells were marked by green fluorescence. Magnification at 200×. $^{*}P < 0.05$, compared to the corresponding gender control group. (A) immunofluorescence of CD11c-positive cells and macrophage cells; (B) the relative number of macrophage cells; (C) the relative number of CD11c-positive cells.

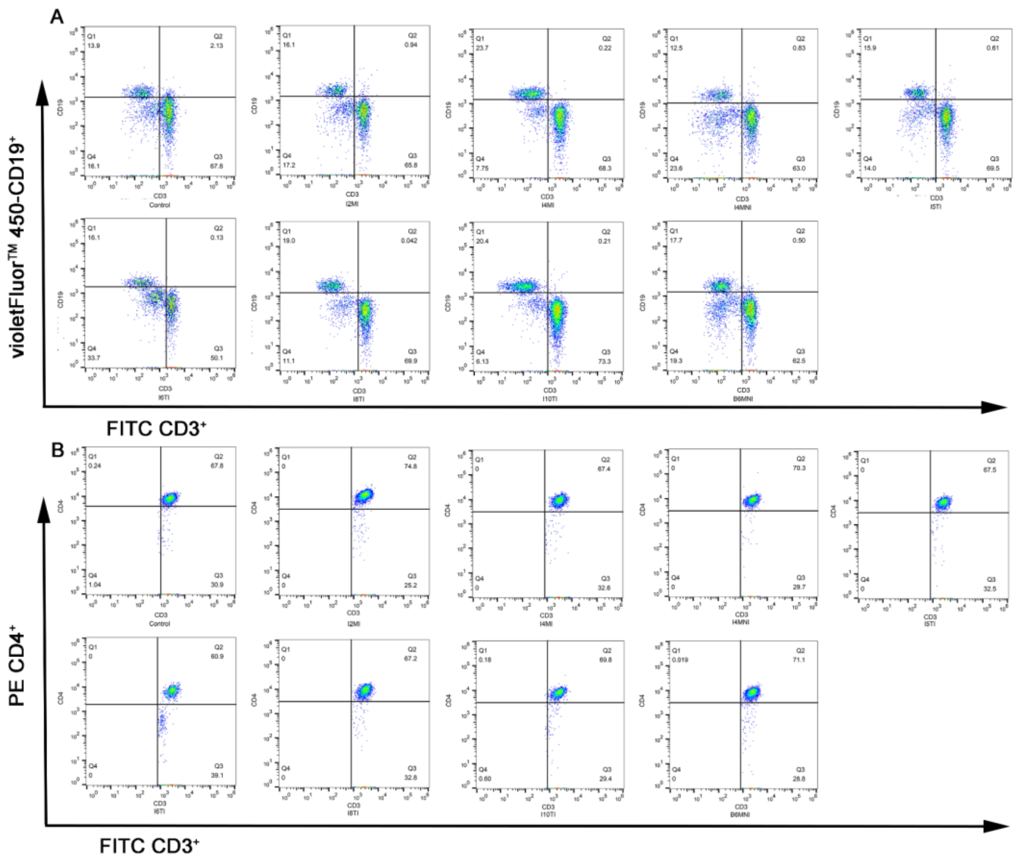

Supplementary Figure 4. Effects of *B. longum* subsp. *infantis* on the percentage of T cells (A, CD3 marker), B cells (A, CD19 marker,), and Th cells (B, CD4 marker) in mesenteric lymph nodes of female mice.

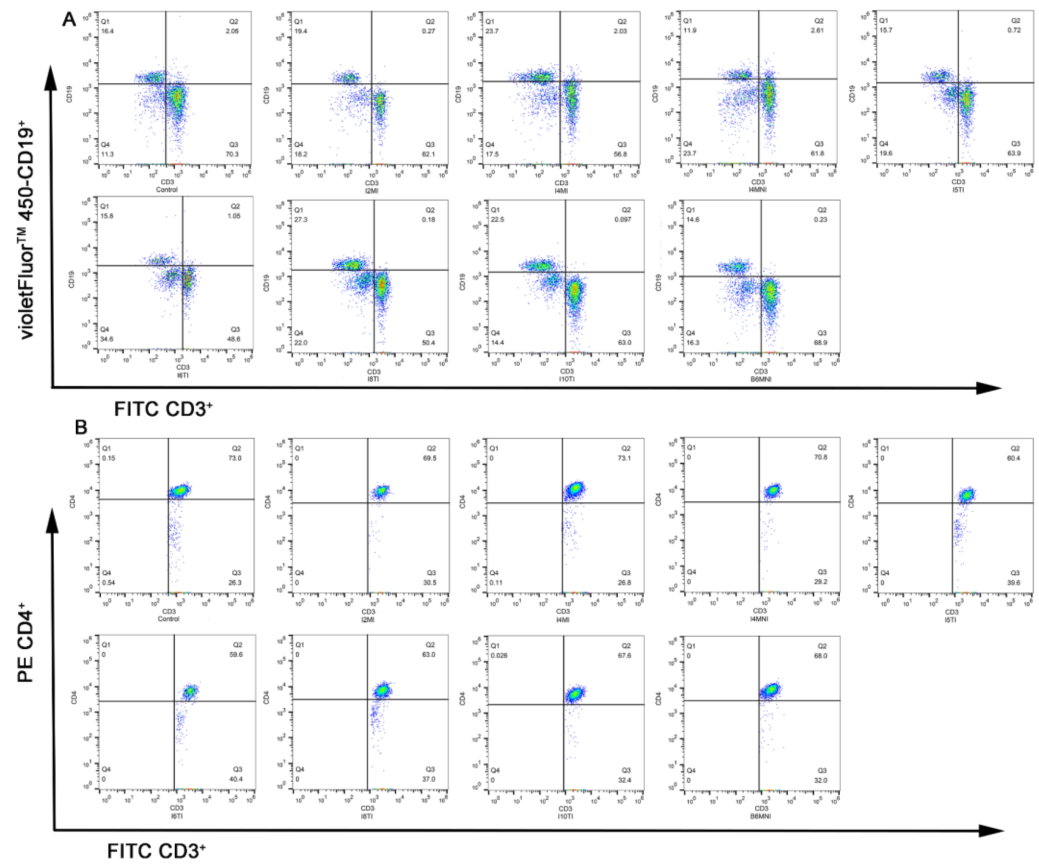

Supplementary Figure 5. Effects of *B. longum* subsp. *infantis* on the percentage of T cells (A, CD3 marker), B cells (A, CD19 marker,), and Th cells (B, CD4 marker) in mesenteric lymph nodes of male mice.

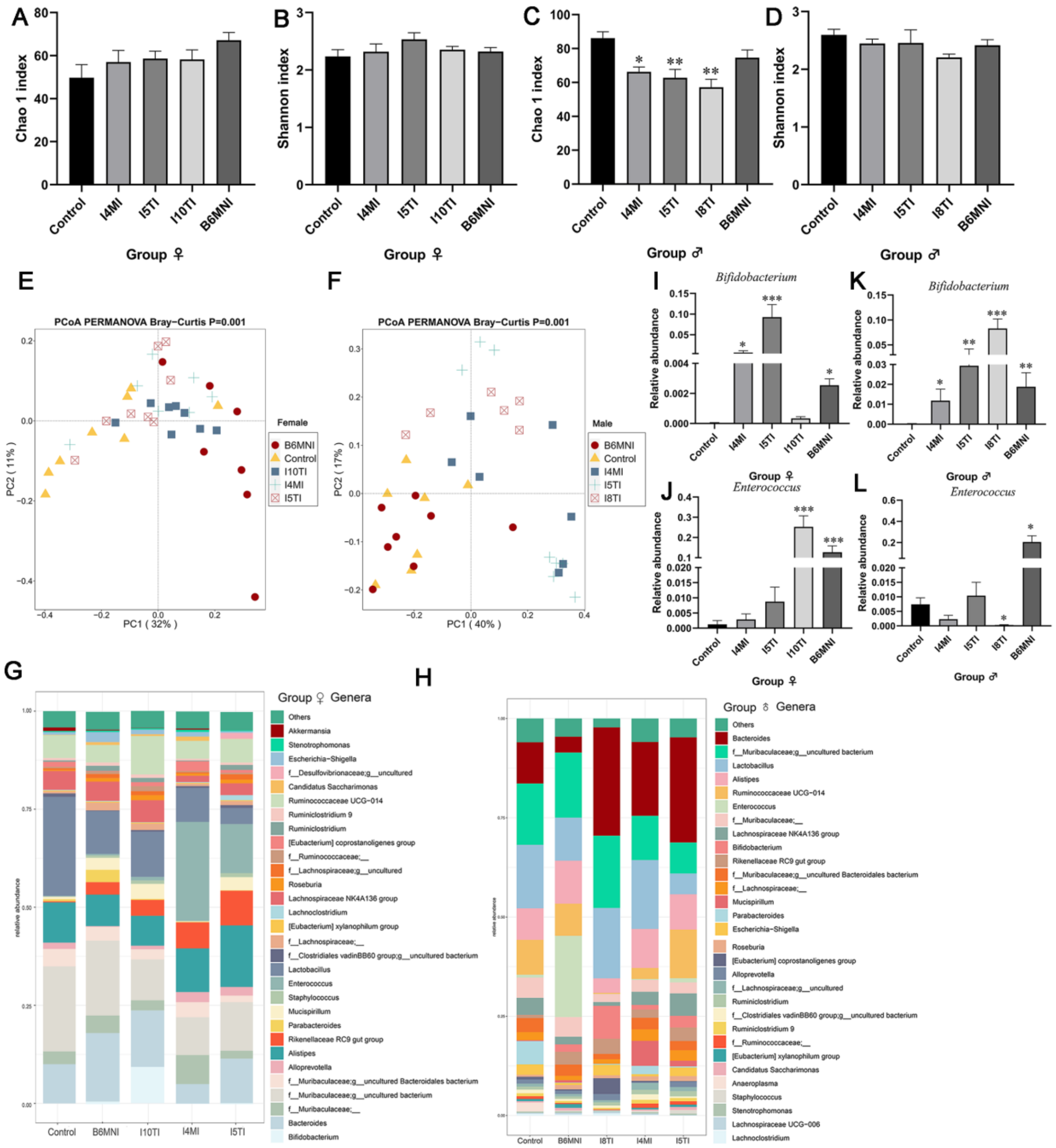

Supplementary Figure 6. Effects of *B. longum* subsp. *infantis* on the diversity of gut microbiota in female and male mice. (A and B) Chao1 and Shannon index of total bacteria in female mice; (C and D) Chao1 and Shannon index in male mice; (E) β-diversity of total bacteria in female mice; (F) β-diversity of total bacteria in male mice; (G) total bacterial stacking plot at genus level in female mice; (H) total bacterial stacking plot at genus level in male mice; (I and J) a significant difference in total bacteria in female mice; (K and L) a significant difference in total bacteria in male mice. $^{*}P < 0.05$, $^{***}P < 0.001$, compared to corresponding gender control group. The mean ± SEM (n = 8 per group) was used to represent the data.

Supplementary Table 1. Primers used in this study

Primer name	Forward (5’-3’)	Reverse (5’-3’)
JAK1	CTCTCTGTCACAACCTCTTCGC	TTGGTAAAGTAGAACCTCATGCG
JAK2	TTGTGGTATTACGCCTGTGTATC	ATGCCTGGTTGACTCGTCTAT
STAT1	TCACAGTGGTTCGAGCTTCAG	GCAAACGAGACATCATAGGCA
STAT6	CTCTGTGGGGCCTAATTTCCA	CATCTGAACCGACCAGGAACT
T-bet	AGCAAGGACGGCGAATGTT	GGGTGGACATATAAGCGGTTC
GATA3	CTCGGCCATTCGTACATGGAA	GGATACCTCTGCACCGTAGC
